# Longitudinal relationships between self-concept for physical activity and neighborhood social life as predictors of physical activity among older African American adults

**DOI:** 10.1186/s12966-017-0523-x

**Published:** 2017-05-22

**Authors:** Allison M. Sweeney, Dawn K. Wilson, M. Lee Van Horn

**Affiliations:** 1 Department of Psychology, University of South Carolina, 1233 Washington Street, 9th Floor, Columbia, SC 29208 USA; 2 Department of Individual, Family, and Community Education, University of New Mexico, Albuquerque, NM 87131 USA

**Keywords:** Physical activity, Neighborhood Social Environment, Self-concept, Older adults

## Abstract

**Background:**

Engaging in regular physical activity (PA) as an older adult has been associated with numerous physical and mental health benefits. The aim of this study is to directly compare how individual-level cognitive factors (self-efficacy for PA, self-determined motivation for PA, self-concept for PA) and neighborhood perceptions of the social factors (neighborhood satisfaction, neighborhood social life) impact moderate-to-vigorous physical activity (MVPA) longitudinally among older African American adults.

**Methods:**

Data were analyzed from a sub-set of older African American adults (*N* = 224, *M*_age_ = 63.23 years, *SD* = 8.74, 63.23% female, *M*_Body Mass Index_ = 32.01, *SD* = 7.52) enrolled in the Positive Action for Today’s Health trial. MVPA was assessed using 7-day accelerometry-estimates and psychosocial data (self-efficacy for PA, self-determined motivation for PA, self-concept for PA, neighborhood satisfaction, neighborhood social life) were collected at baseline, 12-, 18-, and 24-months.

**Results:**

Multilevel growth modeling was used to examine within- and between-person effects of individual-level cognitive and social environmental factors on MVPA. At the between-person level, self-concept (*b* = 0.872, *SE* = 0.239, *p* < 0.001), and neighborhood social life (*b* = 0.826, *SE* = 0.176, *p* < 0.001) predicted greater MVPA, whereas neighborhood satisfaction predicted lower MVPA (*b* = −0.422, *SE* = 0.172, *p* = 0.015). Among the between-person effects, only average social life was moderated by time (*b* = 0.361, *SE* = 0.147, *p* = 0.014), indicating that the impact of a relatively positive social life on MVPA increased across time. At the within-person level, positive increases in self-concept (*b* = 0.294, *SE* = 0.145, *p* = 0.043) and neighborhood social life (*b* = 0.270, *SE* = 0.113, *p* = 0.017) were associated with increased MVPA.

**Conclusions:**

These results suggest that people with a higher average self-concept for PA and a more positive social life engaged in greater average MVPA. Additionally, changes in perceptions of one’s neighborhood social life and one’s self-concept for PA were associated with greater MVPA over 2 years. These factors may be particularly relevant for future interventions targeting long-term change and maintenance of MVPA in older African Americans.

**Trial registration:**

ClinicalTrials.Gov #NCT01025726 registered 1 December 2009.

## Background

The numerous benefits of engaging in regular physical activity (PA) are apparent across the lifespan. Among older adults, PA is associated with numerous physical health benefits, including a significant reduction in risk for all-cause mortality, cardiovascular disease, stroke, and type two diabetes [1–4]. Large-scale longitudinal studies have demonstrated that the benefits of PA are cumulative, such that for every additional 15 min of daily PA, the risk of mortality from any cause decreases by 4% [5]. Furthermore, among older adults PA is associated with a reduced risk of falls and faster recovery from functional limitations, thereby helping older adults to increase the number of years of independent living [1, 6]. Older adults who engage in regular PA also benefit further from a reduced risk of depression [7], improved cognitive functioning [8, 9] including improved memory and hippocampal growth [10], and experience higher levels of well-being [11]. 

Despite the significant physical and mental health benefits, engagement in regular PA decreases with age [12, 13]. In the United States, it is estimated that approximately 28% of adults aged 50 years and older are inactive, with inactivity increasing with age from 25.4% among adults aged 50–64 years, 26.9% among those aged 65–74 years, and 35.3% among those aged ≥75 years [14]. A systematic review of 53 studies from 15 countries revealed that the percentage of adults aged ≥60 years meeting the guidelines for daily PA ranged from 2.4% to 83% with the majority of studies reporting that 40–80% of the samples were failing to meet PA guidelines [15]. In the United States, physical inactivity and the related health consequences are particularly problematic for African Americans, as African Americans are less likely to meet national PA guidelines [16], and are at a significantly higher risk for morbidity and mortality, including higher rates of obesity, type 2 diabetes, and pre-mature death [17, 18]. Health disparities remain a major concern across the lifespan, but may have particularly detrimental effects among older adults, given the higher levels of physical, cognitive, and economic vulnerabilities that accompany older adulthood relative to younger adulthood [19].

Interventions for promoting PA among older adults can be organized from individual-level approaches (e.g., attitudinal, psychological and behavioral factors) through environmental approaches (e.g., neighborhood, built, and social environmental factors) [20]. Whereas some factors, such as PA enjoyment and access to facilities, predict both PA initiation and maintenance among older adults, other factors, such as outcome expectations and action-planning, are more strongly associated with PA initiation than maintenance [21]. Such findings suggest that the determinants of PA among older adults may change over time. At the individual-level, numerous studies have found that self-efficacy, or confidence in one’s capacity to carry out a behavior [22, 23], plays a critical role in PA initiation and maintenance among older adults [25, 26]. Social Cognitive Theory proposes that when people are high in self-efficacy, they are more likely to believe that they can master challenging problems and recover quickly from setbacks [22, 23]. Consistent with this model, among older White adults, high self-efficacy for PA is associated with exercise adherence even after a formal exercise program has finished [24, 25]. Similarly, other studies have found that active older White adults are more likely to be high in self-efficacy for PA than those who are inactive [26]. Furthermore, self-efficacy for PA among White older adults has been associated with exercise adherence across a three-year period [27].

Examining older adult’s motivation for engaging in regular PA has provided additional insight into individual-level factors that promote PA among older adults. Self-Determination Theory posits that behavior should be more stable when people’s motivation is guided by more autonomous (rather than controlled) reasons [28]. That is, people are more likely to engage in PA when they view PA as important, enjoyable, and consistent with their values, rather than an obligation or a means to avoid disapproval from others [29, 30]. Interventions which promote autonomous motivation by allowing participants to have self-directed choice in their PA have proven to be successful for increasing PA across 1 year among older adults [31]. Further underscoring the importance of self-determined motivation for PA among older adults, several studies comprised primarily of older White adults have shown that among individuals coping with chronic illness, those who engage in PA for more autonomous reasons tend to be more physically active [33]. For example, among breast cancer survivors, older women meeting daily PA guidelines had significantly greater autonomous motivation for PA than inactive women [32]. Similarly, among rheumatoid arthritis outpatients, autonomous motivation for PA was positively associated with greater engagement in PA [33]. Furthermore, one prospective study of cardiac rehabilitation outpatients found that autonomous motivation for PA at the end of an exercise rehabilitation program was positively associated with higher levels of PA at follow-up after a six-week period [34].

PA may also depend upon the extent to which PA engagement is viewed as critical to one’s self-concept. Identity theory [35] proposes that the more important any one self-aspect is to one’s overall sense of self, the more likely it is that behaviors associated with that self-aspect will be repeated. Consistent with this model, numerous studies have found that among children and adolescents a positive self-concept for PA predicts future exercise behavior [36, 37], with some studies finding longitudinal effects spanning across 13 years [38]. Similarly, among young adults, those with a positive self-concept for a particular activity (e.g., aerobic exercise) are more likely to engage in that specific activity [39]. However, predictors of PA among adolescents and younger adults may differ from those of older adults. As roles change across the lifespan (e.g., as one transitions from being a parent to a grandparent), individuals’ self-concepts may continue to evolve [40]. However, other roles or activities are not explicitly age-specific and may continue to be a critical component of the self-concept among older adults. For example, among older adults, leisure activities have been found to be especially important for shaping the self-concept [41, 42] and are increasingly mentioned as important descriptors of the self [43]. In light of these findings, a positive self-concept for PA may be especially important in older adulthood; yet, few studies have examined self-concept for PA among older adults.

Social Cognitive Theory proposes that an individual’s behavior interacts with both individual-level cognitive factors and influences from the environment [22, 23]. There is increasing cross-sectional evidence that among older adults various aspects of the neighborhood environment, including perceived neighborhood walkability, safety, and access to recreational facilities, impact walking and PA [44–46]. Importantly, aspects of the physical environment interact with individual-level and social environmental factors to influence PA engagement among older adults. A cross-sectional study of predominantly White community-dwelling adults found that among older adults living in supportive built environments (i.e., high walkability, positive neighborhood aesthetics, access to facilities), those with a positive social environment (i.e., high social support) engaged in greater minutes of MVPA per week [47].

Thus, in addition to perceptions of the physical environment, perceptions of the neighborhood social environment may also impact PA among older adults. Neighborhood social cohesion, or mutual trust and solidarity among neighbors and positive supportive social connections and interactions [48], has been associated with greater levels of walking behavior between neighborhoods among older adults [49]. Other investigators have found that among older adults, individual-level perceptions of social cohesion are positively associated with walking [50]. Similarly, among older adults having a good social network is positively associated with both PA initiation and maintenance [51, 52]. Extending research that has focused on the PA correlates of younger and middle-aged adults [53–55], there is preliminary evidence that individual-level cognitive factors and perceptions of the social environment influence PA engagement among older adults.

Among previous studies examining the role of individual-level and environmental factors on PA among older adults, the majority of research has focused predominantly on White adults [24–27, 32–34, 45, 47, 52]. Although previous interventions have targeted individual-level factors (e.g., behavioral skills training, self-efficacy) as strategies for promoting PA among young and middle-aged African American adults [56, 57], surprisingly little research has addressed the factors that promote PA among older African American adults. Among African American adults, reduced access to facilities, a lack of a safe place to exercise, lack of time, and low motivation are among the most frequently reported barriers to PA [58–62]. Enhanced personal and environmental barriers, coupled with the tendency for PA to decrease with age [12, 13], underscores the need to understand the factors that promote PA initiation and maintenance among older African American adults.

Another key limitation of previous studies of PA among older adults is that relatively little is known about the extent to which the impact of individual-level and environmental factors changes across time. For example, among studies of older adults, several studies have indicated that self-efficacy is important for PA initiation and maintenance [24–27, 63–65]; however, numerous experimental studies have failed to find an association between self-efficacy and PA initiation or maintenance [66–68] (for a review see van Stralen and colleagues, 2009 [21]). Relatedly, recent systematic reviews of self-determined motivation for PA [30] and the role of the built environment [46] both concluded that the majority of extant research has used cross-sectional designs and self-reported PA. Furthermore, to date no study has examined how a positive self-concept for PA relates to accelerometry estimated PA over time among older adults. Taken together, there remain many unanswered questions about how individual-level cognitive and social neighborhood factors may impact PA longitudinally in older adults.

The primary aim of this study was to directly compare how individual-level cognitive factors for PA (self-efficacy for PA, self-determined motivation for PA, self-concept for PA) and neighborhood perceptions of social factors for PA (neighborhood satisfaction, neighborhood social life) impact accelerometer-assessed PA across 24-months among older African American adults. The present study uses data collected from participants in the Positive Action for Today’s Health (PATH) trial [69–71], which includes measures of self-efficacy for PA, self-determined motivation for PA, self-concept for PA, neighborhood satisfaction, neighborhood social life, and accelerometer-assessed MVPA from baseline through 24 months. The hierarchical nature of this data allows us to examine both between- and within-person effects. We hypothesized that between-person mean differences in self-efficacy for PA, self-determined motivation for PA, self-concept for PA, neighborhood satisfaction and social life would be positively associated with greater MVPA. Additionally, we hypothesized that the relationship between MVPA and the between-person effects would remain constant across time (i.e., no significant interactions with time). Finally, we examined whether within-person changes in the predictors were associated with changes in MVPA as exploratory analyses.

## Methods

### Design and setting

The PATH study randomized communities to receive an environmental intervention for increasing perceptions of safety and access to neighborhood-based physical activity among African Americans [69–71]. Three communities in South Carolina were selected based on census tract level information and were randomized to receive one of three interventions: a police-patrolled-walking program with a social marketing intervention, a police-patrolled walking program only, or a no-walking general health education program. Although the larger trial led to greater neighborhood trail walking, there were no between-group differences in accelerometer-estimates of MVPA in the full intervention community. Measures were collected at baseline, 12, 18, and 24 months. To measure MVPA, participants wore accelerometers for seven consecutive days. Additionally, at each time point participants completed a series of psychosocial measures, including self-concept for PA, self-determined motivation for PA, self-efficacy for PA, neighborhood satisfaction, and neighborhood social life. The study was approved by the Institution Review Board at the University of South Carolina. All participants signed informed consent prior to participating in the study and received monetary compensation in the form of a US $20 gift card at each assessment period, with the exception of the 24-month assessment when participants received a US $40 gift card.

### Participants

Two recruitment strategies were used in the PATH trial. First, participants were recruited via telephone calls and letters from a list of households in specified census tracts in each targeted community provided by the University of South Carolina Survey Laboratory. Of the 1986 households contacted, 1216 were reached, 581 declined, 635 individuals were invited to participate, and 231 enrolled in the study. As a second recruitment method, 203 volunteers were recruited using flyers, ads placed in local newspapers, and posters placed in churches, schools and local businesses [69–71]. Approximately 54% of the final sample was recruited using the random list of households and 46% was recruited through volunteer advertisements. The inclusion criteria for the study included: 1) African American (three of four grandparents of African heritage), 2) 18 years or older, 3) had no plans to move in the next two years, 4) had no medical condition that would prevent them from engaging in regular PA, and 5) had controlled blood pressure (systolic <180 mmHg systolic and diastolic <110 mmHg) and blood sugar levels (non-fasting <300 mg/dl and fasting <250 mg/dl). Participants were excluded if they indicated that they could not safely participate in moderate PA. A total of 434 adults were enrolled in the study (with age ranging from 18 to 85 and a median age of 51 years). Seventeen participants did not provide accelerometer-assessed MVPA at any of the time points, and were therefore excluded from data analysis. Given that the present study focuses specifically on older adults, we conducted a median split and included data from adults who were 51 years or older, yielding a final sample size of 224. The decision to include adults over the age of 50 is consistent with the inclusion criteria used in systematic reviews of PA among older adults [21].

### Data collection and measures

#### Actical accelerometer assessments

At each time point, Actical devices were used to assess MVPA over seven consecutive days (Mini-Mitter, Bend, OR). Acticals are small electronic devices that assess both intensity and frequency of movement to index daily minutes of light, moderate, and vigorous physical activity. Whereas accelerometers detect movement on uni- and bi-directional planes, Acticals detect movement omnidirectionally, and therefore provide a more sensitive measure of PA [72]. Acticals have acceptable test re-test reliability coefficients for MVPA ranging from 0.85–0.90 [73]. Participants were instructed to wear the Actical on their right hip at all times, excluding sleep and occasions when the Actical could get wet. Activity was categorized as MVPA if counts per minutes were at least 1075, which was based on a calibration study conducted with a sample similar to the PATH trial [74]. MVPA scores index daily minutes of PA.

#### Self-concept for PA

Participants completed the 10-item Self Concept and Motivation to Exercise Scale to assess their health self-concept concerning the importance of increasing PA and motivation to change PA (e.g., “I am the type of person who likes to exercise daily”; “Exercising regularly is a very important part of my everyday life”) [75, 76]. Items were answered on a 6-point scale ranging from one (*Strongly Disagree*) to six (*Strongly Agree*). The scale demonstrated acceptable reliability at all time points (*α* = 0.83–0.87) and past studies have demonstrated construct validity among African American populations [75, 76].

#### Self-determined motivation for PA

Participants completed a brief 8-item version of the Behavioral Regulation in Exercise Questionnaire assessing their reasons for engaging in exercise (e.g., “I enjoy my exercise sessions”; “I think it’s important to make the effort to exercise regularly”; adapted from Mullan and Markland [77]). Items were answered using a 5-point scale ranging from one (*Not True of Me*) to five (*Very True of me*). The scale demonstrated acceptable reliability at all time points (*α* = 0.72–0.75) and past studies have demonstrated construct validity among African American populations [78].

#### Self-efficacy for PA

Participants completed the 16-item Self-Efficacy for Exercise Questionnaire to assess their confidence in their ability to exercise when faced with potential barriers (e.g., “I am confident I could exercise over the next 6 months when tired”; “I am confident I could exercise over the next 6 months during bad weather”; adapted from Garcia and King [79]). Items were answered using an 11-point scale ranging from 0% confident to 100% confident. The scale demonstrated acceptable reliability at all time points (*α* = 0.95–0.96) and past studies have demonstrated acceptable internal consistency and test-retest reliability [79].

#### Neighborhood satisfaction

Participants completed the 26-item Neighborhood Environment Walkability Scale [80]. Six items were used to compute the Neighborhood Satisfaction subscale to assess participants’ general satisfaction with their neighborhood (e.g., “How satisfied are you with how easy and pleasant it is to walk in your neighborhood?”; “How satisfied are you with how many friends you have in your neighborhood?”) Items were answered on a 5-point scale ranging from one (*Strongly Dissatisfied*) to five (*Strongly Satisfied*). The scale demonstrated acceptable reliability at all time points (*α* = 0.78–0.83) and past studies have demonstrated good internal consistency and test-retest reliability [80].

#### Neighborhood social life

Participants completed 9-items from the Neighborhood Social Interaction scale (adapted from Elliot et al. [81]). Items assessed how often participants had engaged in informal social activities in their neighborhood (e.g., “Stopped and talked with a neighbor”). The scale demonstrated acceptable reliability at all time points (*α* = 0.80–0.86) and past studies have demonstrated acceptable internal consistency [81].

#### Statistical analyses

To account for differences in scaling across the five predictors, individual items for each of the five scales were z-scored. The z-scored values for each scale were summed, and then z-scored again. A square root transformation was applied to MVPA to normalize a skewed distribution. Accelerometer data were missing for 4.01% of participants at baseline and for 19.64–26.34% at each of the follow-up time points. A compliance criterion of one or more 4-h intervals was used and combined with multiple imputation, a robust technique for accounting for missing data, that is considered appropriate for accelerometry and longitudinal data [82–84]. The MICE package in R [85] was used to generate 20 imputations (for further discussion of this approach see our previous publication [70]). One imputation was randomly selected for conducting the present analyses.

Multilevel growth modeling [86] was used to account for the hierarchical structure of the data. To explore statistical assumptions associated with multilevel modeling, the residuals of the random effects were examined in the model. Plots of the standardized Level − one studentized residuals against their normal scores showed a linear association, suggesting relative normality and no extreme outliers. Additionally, plots of the residuals against the predicted values of MVPA showed no signs of significant heteroscedacicity.

Each predictor (self-determined motivation for PA, self-efficacy for PA, self-concept for PA, neighborhood satisfaction, and neighborhood social life) was parsed into separate between- and within-person components. Between-person scores were calculated by averaging an individual’s score on a predictor across time and within-person scores were calculated by subtracting each person’s score at each time point from his or her personal mean. The slopes for the between-person component indexes the magnitude of the relationship between individuals’ mean score on a predictor and their mean level of MVPA. Time was centered such that (baseline = −1, 12 months = 0, 18 months = .5, 24 months = 1), such that a 1-unit change in time corresponds to the passage of 1 year. Additionally, the models tested for interactions between time and the between-person mean scores. A non-significant interaction would indicate that the relationship between participant’s mean level on a predictor and MVPA remained stable across the 24-month follow-up period.

To aid convergence, only the intercept and time were permitted to vary as random effects. Age, sex (0 = male, 1 = female), baseline body mass index (BMI), and baseline annual income (0 = < $10,000, 1 = $10,000–24,000, 2 = > $24,000) were included as covariates. Additionally, two dummy coded community variables were included as covariates to account for intervention effects from the larger trial. Grand-mean centering was applied to age and BMI.

A hierarchical approach was used to analyze the data, such that model one includes only the effects of the covariates and time. Model two includes the addition of the between- and within-person factors. Finally, model three includes the addition of the interaction terms between the between-person factors and time. To examine whether the addition of the between- and within-person factors and the interaction terms significantly contributed to the prediction of MVPA, likelihood ratio tests were conducted to compare the change in the residual between model 1 and 2, and between model 2 and 3. By comparing the change in the residual, likelihood ratio tests allows one to examine whether there is a significant increase in the total variance accounted for in a given model.

## Results

### Descriptive statistics

Baseline characteristics of the sample are presented in Table 1. The sample was African American and predominantly female (68.30%), obese (*M*_BMI_ = 32.01, *SD* = 7.52), ranged in age from 51 to 85 years (*M* = 63.23, *SD* = 8.74), and had a mean annual household income that was less than or equal to $25,000.

**Table 1:** Baseline characteristics of path sample (*N* = 224)

Valiable	
Gender	
Male	71
Female	153
Age (years)	
Mean (SD)	63.23 (8.74)
50–55	52
56–60	44
61–65	46
66–70	27
71–75	33
76–85	22
Marital status	
Married	63
Separated	27
Divorced	31
Widowed	75
Never married	24
Unmarried couple	4
Employment	
Working	72
Laid off/unemployed	28
Retired	97
Disabled	22
Homemaker	4
Student	1
Education	
< HS degree	67
HS degree/GED	78
Some college/technical training	45
College degree	12
Graduate/professional degree	22
Income	
< US$10,000	54
US$10,000–24,000	88
> US$24,000	82
BMI	
Mean (SD)	32.01 (7.52)
< 25	32
25- < 30	69
> 30	123

### Correlations

Table 2 presents the unstandardized means and standard deviations for self-efficacy for PA, self-determined motivation for PA, self-concept for PA, neighborhood social life, neighborhood satisfaction, and MVPA at all time points. Self-concept scores were moderately correlated across all time points with self-determined motivation (*r*s = 0.51–0.64), and self-efficacy (*r*s = 0.52–0.59), but not at the magnitude of collinearity. Self-concept scores had a small correlation with neighborhood social life (*r*s = −0.29 - 0.09) and neighborhood satisfaction (*r*s = 0.14–0.29). All other bivariate correlations between predictors across time points were less than or equal to *r* = 0.48.

**Table 2:** Means standard deviations for the individual-level cognitive and social environment factors across time

	Baseline		12 months		18 months		24 months	
Valiables	*M*	*SD*	*M*	*SD*	*M*	*SD*	*M*	*SD*
Self-Determined Motivation for PA	3.39	0.81	3.41	0.77	3.35	0.28	3.38	0.28
Self-Concept for PA	4.20	0.98	4.11	0.96	4.06	0.96	3.95	1.00
Self-Efficacy for PA	61.78	22.54	56.63	23.39	53.75	22.28	50.32	24.20
Neighborhood Satisfaction	3.84	0.37	3.69	0.85	3.76	0.83	3.87	0.75
Neighborhood Social Life	9.13	5.85	9.76	5.78	9.81	6.47	10.32	7.29
MVPA	17.06	26.60	24.48	32.14	25.38	41.54	17.63	30.63

Raw means are presented in Table 2. Analyses were conducted on z-scored scale values and square root transformed MVPA

### Model ICCs

Intercept-only models (i.e., no predictors) were conducted for all study variables to examine the degree of variance attributable to the within- and between-person levels. From these models, intraclass correlation coefficients (ICC) were computed, which provide an index of the proportion of variance associated with between-person differences. The ICCs indicated that between 43.91 to 53.62% of the variance was attributable to between-person differences. Thus, the use of multi-level modeling was deemed appropriate [87].

### Between and within-person effects on MVPA

A hierarchical approach was used to examine whether the inclusion of the covariates (model 1), the between and within-person effects (model 2), and the time*between-person interaction effects (model 3) improved the prediction of MVPA. Models 1–3 yielded *R* ^*2*^ values of 0.153, 0.240, and 0.249, respectively. To examine whether the addition of the between- and within-person factors (model 2) and the interaction terms (model 3) significantly contributed to the prediction of MVPA, likelihood ratio tests were conducted to compare the change in the residual between the models. The inclusion of the between- and within-person factors in model 2 yielded a significant reduction in the −2 Residual Log Likelihood relative to model 1, *χ* ^*2*^ (1) = 20.77, *p* < 0.001. The inclusion of the interaction terms yielded a small and non-significant reduction in the residual relative to model 2, *χ* ^*2*^ (1) = 0.29, *p* = 0.59. The results of the likelihood ratio tests revealed that including the individual-level cognitive and environmental factors significantly improved the prediction of MVPA relative to a covariates-only model. Although collectively the inclusion of the between-person*time interactions did not significantly improve the prediction of MVPA, we present the full results of model 3 to examine whether any of the individual between-person*time interaction effects significantly predict MVPA (see Table 3).

**Table 3:** Multilevel growth models predicting MVPA

					*Lower*	*Upper*
Predictors	*b*	*SE*	*t*	*p*	*95% CI*	*95% CI*
Fixed effects						
Intercept	4.171	0.329	12.693	<.001	3.526	4.816
Time	0.115	0.102	1.132	0.258	−0.084	0.314
Body mass index	−0.014	0.016	−0.926	0.355	−0.045	0.016
Age	−0.0947	0.014	−6.807	<.001	−0.121	−0.067
Female	−0.983	0.271	−3.622	<.001	−1.518	−0.448
Income	−0.117	0.158	−0.740	0.460	−0.429	0.195
Community Dummy Code 1 (Walking Only)	0.425	0.291	1.461	0.146	−0.148	0.998
Community Dummy Code 2 (Full Intervention)	0.602	0.285	2.115	0.036	0.041	1.163
Within-person change						
Self-Determined Motivation for PA	−0.147	0.126	−1.165	0.245	−0.395	0.101
Self-Efficacy for PA	−0.092	0.121	−0.759	0.448	−0.329	0.146
Self-Concept for PA	0.294	0.145	2.026	0.043	0.009	0.579
Neighborhood Satisfaction	−0.212	0.117	−1.820	0.069	−0.442	0.017
Neighborhood Social Life	0.270	0.113	2.392	0.017	0.048	0.492
Between-person difference						
Mean Self-Determined Motivation for PA	−0.151	0.231	−0.655	0.513	−0.606	0.303
Mean Self-Efficacy for PA	−0.003	0.199	−0.017	0.987	−0.395	0.388
Mean Self-Concept for PA	0.872	0.239	3.644	<.001	0.400	1.344
Mean neighborhood Satisfaction	−0.422	0.172	−2.451	0.015	−0.762	−0.083
Mean neighborhood Social Life	0.826	0.176	4.702	<.001	0.479	1.172
Between-person interactions						
Time • Mean Self-Determined for PA	−0.345	0.188	−1.839	0.066	−0.714	0.023
Time • Mean Self-Efficacy for PA	−0.059	0.165	−0.361	0.718	−0.382	0.264
Time • Mean Self-Concept for PA	0.213	0.201	1.057	0.291	−0.183	0.608
Time • Neighborhood Satisfaction	0.084	0.142	0.593	0.554	−0.194	0.362
Time •Neighborhood Social Life	0.361	0.147	2.456	0.014	0.072	0.649
Random effects						
Intercept	1.317				1.223	1.545
Time	0.215				0.068	0.682
Residual	2.118					

Model *R* ^2^ = 0.49

As seen in Table 3, being younger in age^1^ and being male were associated with greater levels of MVPA. Additionally, being assigned to the full intervention community (relative to the control community) was associated with greater MVPA. There was no significant fixed effect of time, income, or BMI. At the within-person level, only changes in neighborhood social life and changes in self-concept predicted increased MVPA. At the between-person level, self-concept and neighborhood social life predicted greater MVPA, whereas neighborhood satisfaction predicted lower MVPA. Among the between-person effects, only average social life was moderated by time. The positive sign of this interaction indicates that people with a higher average neighborhood social life engaged in greater MVPA than people with a lower social life, with the strength of this association increasing across time. Conversely, the impact of average neighborhood satisfaction and average self-concept remained relatively stable across time, as time did not significantly moderate the effect of these factors. At the within-person level, neighborhood social life and self-concept predicted increased MVPA, indicating that positive increases in self-concept and neighborhood social life were associated with increased MVPA.

## Discussion

The results of this study indicated that that people with a higher average self-concept for PA engaged in greater MVPA than people with a lower average self-concept for PA. Furthermore, people with more positive perceptions of their neighborhood social life engaged in greater MVPA than people with less positive perceptions. Additionally, changes in perceptions of one’s neighborhood social life and changes in one’s self-concept for PA were associated with increased MVPA. Thus, when compared directly with other individual-level cognitive factors (self-efficacy for PA, self-determined motivation for PA), having a positive self-concept for PA and a positive social life were significant predictors of longitudinal MVPA among older African American adults.

In contrast to previous studies, which have found positive associations between PA and self-determined motivation [32–34] and self-efficacy [24–27], the present study demonstrated that being relatively high or low on these factors did not predict MVPA across time in older African Americans. Additionally, in the present study we did not observe any significant within-person effects of self-determined motivation or self-efficacy on MVPA. Previous studies of older adults have indicated a positive association between self-efficacy and PA through cross-sectional designs [26], but longitudinal studies have found evidence of both growth [88] and decline [89] of self-efficacy across time. Similarly, the majority of past studies of self-determined motivation and PA have used cross-sectional designs [30]. Changes in personal and environmental barriers across time may differentially influence one’s perceived competence and motivation for engaging in PA; as a result, self-efficacy and self-determined motivation for PA may be relatively less impactful on long-term PA maintenance. Alternatively, to the extent that one’s self-concept helps to organize and guide one’s actions [90], it follows that a positive self-concept for PA will be a relatively robust predictor of PA maintenance. Taken together, the present findings underscore the importance of complementing cross-sectional studies with longitudinal designs to clarify how the relationship between individual-level cognitive factors and PA maintenance change across time among older adults.

In the present study we found that among the three communities included in the PATH trial, a more positive neighborhood social life predicted greater MVPA across 24 months. In a previous study from the PATH trial [91], we found that that across 12 months participants in the full intervention community of the PATH trial engaged in more trail walking outside of their neighborhood when perceptions of neighborhood social life were low. Although this finding may appear somewhat inconsistent with the present results, the present study varies on a number of critical dimensions from the previous study [91], including differences in PA intensity (walking vs. MVPA), PA measurement (self-report vs. accelerometry estimates), and measurement length (12 months vs 24 months). Taken together, we conclude that when perceptions of the neighborhood social life are low people are more likely to pursue opportunities for PA outside of their immediate neighborhood (such as joining a walking group on a community trial), but overall when neighborhood social life is high greater adherence to MVPA may occur within the context of one’s neighborhood.

Surprisingly, in the present study we found that greater average neighborhood satisfaction predicted lower MVPA. Whereas past studies have found positive effects of neighborhood satisfaction on PA among older adults [92], such studies have tended to be cross-sectional and have focused on older White adults. Importantly, past studies have documented mixed results regarding the relationship between specific aspects of the environment and physical activity among older adults [93]. To further clarify the relationship between neighborhood satisfaction, social life, and MVPA, future research should examine how different intensities of PA, both within and outside of one’s immediate neighborhood, varies across racial, ethnic, and socio-economic status groups.

The present findings highlight a positive neighborhood social life as a key predictor of PA among older African Americans. Much of the extant literature on PA among older adults has focused on the role of the physical environment, including factors such as perceptions of safety, neighborhood walkability, and proximity of recreational facilities [44–46, 94]. Relatively fewer studies have examined the role of the social environment among older adults, with much of the previous research focusing on other aspects of the social environment, including the role of social support [26, 47, 95, 96], social cohesion [49, 50] and neighborhood or social involvement [97, 98] as predictors of PA. Building on previous prospective work of social environmental factors [99], and cross-sectional studies [26, 48, 49, 95–98], the present findings provide further evidence for the longitudinal impact of a positive neighborhood social life on objectively-assessed PA among older adults.

Future research is needed to test how positive changes in self-concept and neighborhood social life can be further integrated into PA interventions. For example, past studies have found that helping people to make deliberate connections between their values and a target behavior is an effective behavior change strategy for adoption of health behaviors [100], with past studies finding positive effects of such programs among African Americans [56]. Furthermore, there is evidence that focusing on one’s values promotes an expanded sense of one’s self-concept [101]. Future research could build upon this approach by examining how focusing on one’s core values could be used to facilitate positive changes in one’s self-concept for PA for long-term change and maintenance. Additionally, future interventions should consider how to develop community-based programs to promote social support, cohesion, and positive social opportunities for engaging in PA within neighborhoods.

By incorporating a longitudinal design, an objectively-assessed measure of PA, and a sample of older African American adults, the present study addresses some of the key methodological limitations of past research. Furthermore, relatively few studies have examined how individual-level cognitive factors and neighborhood perceptions impact longitudinal MVPA among predominantly older obese African American adults. In the United States, African American adults have the highest rate of obesity [102], and a higher rate of physical inactivity relative to White adults [103, 104]. Interestingly, whereas our previous work tested for intervention effects among the full PATH trial sample (*N* = 417, ages 18–85) [70], by focusing specifically on older adults in the PATH trial, the present study revealed a treatment effect that was not reported in our previous research. These findings suggest that the full intervention was particularly beneficial for increasing MVPA among relatively older participants. Taken together, the present results provide valuable insight into the individual and social environmental factors that predict longitudinal MVPA among older African Americans in the PATH trial.

There are several limitations to the present study that should be acknowledged. One limitation of the present research is that the generalizability of the present findings beyond African Americans remains unclear. Future research is needed to confirm that the positive effect of self-concept for PA and neighborhood social life generalize to other racial and ethnic groups of older adults. Another limitation of the present research is that the present analyses were conducted on a single imputation, which may yield less reliable estimates than a combined multiple imputation approach. A third limitation is that the trial recruited a mixture of randomly selected and volunteer participants. By including volunteer participants the trial may have inadvertently selected some participants with a higher level of motivation.

## Conclusions

This study expands on past research by demonstrating that among older African American adults, those with a higher average positive self-concept for PA engaged in greater MVPA than those with a low average self-concept. This is one of the first studies, to date, to examine how self-concept for PA relates to longitudinal objective assessments of MVPA among older adults. Additionally, positive increases in one’s perceived neighborhood social life and in one’s self-concept for PA were associated with increases in MVPA over time. Future interventions should consider how positive changes in self-concept and the neighborhood social environment can be further integrated into PA interventions in older at-risk adults.

## Abbreviations

BMI: Body mass index
ICC: Intraclass correlation coefficients
MVPA: Moderate to vigorous physical activity
PA: Physical activity

## Footnotes

1 To examine further how the observed effects were related to age, we ran additional analyses to test whether age moderated any of the between-person effects. At the between-person level, only average self-determined motivation was moderated by age, *B* = −0.060, *SE* = 0.026, *p* = 0.026, 95% *CI* = −0.112, −0.007.
